# Magnetic scattering in the simultaneous measurement of small-angle neutron scattering and Bragg edge transmission from steel^1^

**DOI:** 10.1107/S1600576716013133

**Published:** 2016-09-16

**Authors:** Yojiro Oba, Satoshi Morooka, Kazuki Ohishi, Nobuhiro Sato, Rintaro Inoue, Nozomu Adachi, Jun-ichi Suzuki, Toshihiro Tsuchiyama, Elliot Paul Gilbert, Masaaki Sugiyama

**Affiliations:** a Research Reactor Institute, Kyoto University, 2 Asashiro-nishi, Kumatori, Osaka 590-0494, Japan; b Department of Aerospace Engineering, Tokyo Metropolitan University, 6-6 Asahigaoka, Hino, Tokyo 191-0065, Japan; c Comprehensive Research Organization for Science and Society, 162-1 Shirakata, Tokai, Ibaraki 319-1106, Japan; d Department of Mechanical Engineering, Toyohashi University of Technology, 1-1 Hibarigaoka, Tempaku-cho, Toyohashi, Aichi 441-8580, Japan; e Department of Materials Science and Engineering, Kyushu University, 744 Motooka, Nishi-ku, Fukuoka, 819-0395, Japan; f Bragg Institute, Australian Nuclear Science and Technology Organisation, Locked Bag 2001, Kirrawee DC, NSW 2232, Australia

**Keywords:** small-angle neutron scattering, Bragg edge transmission, steel, magnetic scattering

## Abstract

Pulsed neutron sources enable the simultaneous measurement of small-angle neutron scattering (SANS) and Bragg edge transmission. This simultaneous measurement is useful for microstructural characterization in steel. Since most steels are ferromagnetic, magnetic scattering contributions should be considered in both SANS and Bragg edge transmission analyses. An expression for the magnetic scattering contribution to Bragg edge transmission analysis has been derived. The analysis using this expression was applied to Cu steel. The ferrite crystallite size estimated from this Bragg edge transmission analysis with the magnetic scattering contribution was larger than that estimated using conventional expressions. This result indicates that magnetic scattering has to be taken into account for quantitative Bragg edge transmission analysis. In the SANS analysis, the ratio of magnetic to nuclear scattering contributions revealed that the precipitates consist of body-centered cubic Cu_0.7_Fe_0.3_ and pure Cu, which probably has 9R structure including elastic strain and vacancies. These results show that effective use of the magnetic scattering contribution allows detailed analyses of steel microstructure.

## Abstract 2

A technique for the analysis of magnetic scattering has been developed, where small-angle neutron scattering and Bragg edge transmission measurements are performed simultaneously. This technique is shown to provide crystallographic information for ferrite crystallites and nanostructural information for precipitates in steel.

## 1. Introduction   

Small-angle neutron scattering (SANS) and neutron diffraction (ND) are powerful techniques to quantitatively investigate microstructures in steels. SANS characterizes precipitates and inclusions (Große *et al.*, 2000 ▸; Ohnuma *et al.*, 2009 ▸; Seong *et al.*, 2010 ▸), while ND provides information about the crystal structure of the steel matrix (Harjo *et al.*, 2001 ▸; Tomota *et al.*, 2003 ▸). A new measurement technique that utilizes SANS and ND simultaneously has been recently developed using new-generation pulsed neutron sources such as SNS and J-PARC (Oba *et al.*, 2015 ▸). In the technique presented here, Bragg edge transmission analysis was combined with SANS instead of ND to overcome problems associated with the difference in experimental settings between SANS and ND. Since the Bragg edge transmission is observed as the decrease in neutron transmission due to Bragg diffraction, it provides identical information to ND (Sato *et al.*, 2011 ▸). Bragg edge transmission analysis needs only the neutron transmission spectrum, which can be simply measured using a transmission monitor placed just after the sample. In using time-of-flight SANS (TOF-SANS), the transmission spectra are always measured during general experimental procedures (Heenan *et al.*, 1997 ▸). Therefore, using pulsed neutron sources, SANS measurements can be smoothly extended to simultaneous measurement of SANS and Bragg edge transmission.

In conventional Bragg edge transmission analyses, magnetic scattering contributions have been ignored. However, since most steels are ferromagnetic and generate significant magnetic scattering, the magnetic scattering contribution should be considered. The information obtained from magnetic scattering is meaningful in analysis of magnetic structures. In addition, combined analysis of nuclear scattering and magnetic scattering contributions can give more detailed structural information. Therefore, magnetic scattering in the simultaneous measurement of SANS and Bragg edge transmission was investigated in this study.

## 2. Magnetic scattering in Bragg edge transmission from steel   

The neutron attenuation coefficient μ(λ) of matter at the neutron wavelength λ is expressed as follows (Sato *et al.*, 2011 ▸):
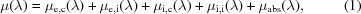
where μ_e,c_(λ), μ_e,i_(λ), μ_i,c_(λ), μ_i,i_(λ) and μ_abs_(λ) represent contributions due to elastic coherent scattering, elastic incoherent scattering, inelastic coherent scattering, inelastic incoherent scattering and absorption, respectively. The term μ_e,c_(λ) gives the Bragg edge transmission. The contributions of the other terms are not significant between λ = 0.2 and 0.4 nm and can be calculated from the chemical composition (Granada, 1984 ▸; Sato *et al.*, 2011 ▸).

From neutron diffraction theory, μ_e,c_(λ) can be described as follows:
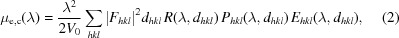
where *V*_0_, *F_hkl_* and *d_hkl_* are the unit-cell volume, crystal structure factor and lattice spacing of {*hkl*}, respectively. The functions *R_hkl_*(λ, *d_hkl_*), *P_hkl_*(λ, *d_hkl_*) and *E_hkl_*(λ, *d_hkl_*) are the resolution function, preferred orientation function and primary extinction function, respectively. The effect of the crystallite size is included in *E_hkl_*(λ, *d_hkl_*) and is apparent in the jump height of the Bragg edge transmission (Sato *et al.*, 2011 ▸). The function *P_hkl_*(λ, *d_hkl_*) indicates the crystallographic texture of the matrix and mainly affects the shape of each Bragg edge transmission (Sato *et al.*, 2011 ▸).

**Figure 1:**
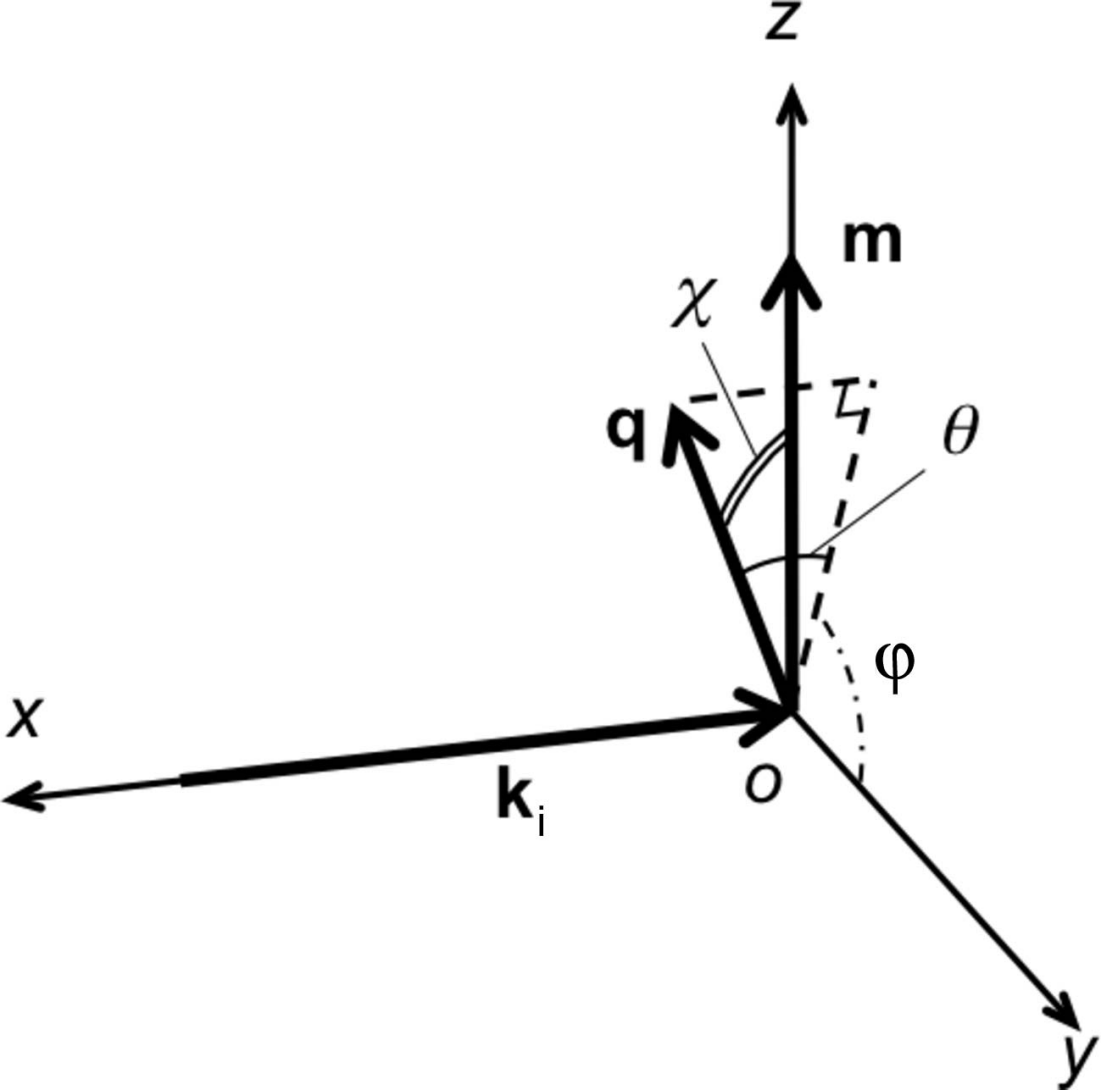
Coordinate system showing the relationship between the vector magnetization **m** and the unit scattering vector **q**. The vector **k**_i_ denotes the direction of the incident neutrons.

To include the magnetic crystal structure factor *F*_m_, equation (2) can be modified to include the summation of the nuclear and magnetic scattering contributions (Squires, 1996 ▸). In ferromagnetic materials, there is no difference in *V*_0_, *d_hkl_*, *R_hkl_*(λ, *d_hkl_*) and *P_hkl_*(λ, *d_hkl_*) between the nuclear and magnetic scattering contributions. In general, unsaturated magnetization causes magnetic domains with domain sizes different from the crystallite sizes; however, in the present study, since the magnetization of the samples is fully saturated in a high magnetic field (SANS setting), the possible effects of magnetic domains can be ignored. Consequently, the value of *E_hkl_*(λ, *d_hkl_*) is unchanged and it is only necessary to consider the changes in *F_hkl_*. As a result, magnetic scattering is dealt with by substitution for *F_hkl_* using the following equation:
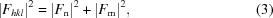
where *F*_n_ is the nuclear crystal structure factor. A detailed explanation of the calculation of *F*_m_ is provided by Squires (1996 ▸). The factor *F*_m_ is dependent on the magnitude and the orientation of the vector magnetization **m** with respect to the unit scattering vector **q**. Fig. 1 ▸ shows the relationship of **m**, **q** and the direction of the incident neutrons **k**_i_. The sample is located at the origin, the magnetization is aligned along the *z* axis and the incident neutrons pass along the *x* axis. The value of |*F*_m_|^2^ is then proportional to the component |**m**_perp_|^2^ given by
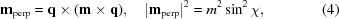
where χ is the angle between **m** and **q**. The value *m* is the magnitude of the magnetization. In Fig. 1 ▸, half the scattering angle θ coincides with the angle between **q** and the *yz* plane. In the Bragg edge transmission spectra, the angle of **q** varies from θ = 0 to π/2 as λ becomes longer according to Bragg’s law (λ = 2*d_hkl_*sinθ). Bragg edge transmission analysis includes the orientation average of the diffraction with the azimuth angle φ in the *yz* plane. Using θ and φ, χ is written as
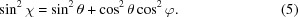
Then, |**m**_perp_|^2^ in the SANS setting is described as follows:
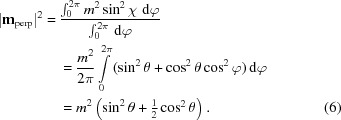
Equation (6) indicates that |**m**_perp_|^2^ in the Bragg edge transmission is represented by a function of θ in the SANS setting. According to equation (6), the magnetic scattering is proportional to *m* ^2^/2 at θ = 0 and *m* ^2^ at θ = π/2.

*F*_m_ is also proportional to the magnetic form factor, which is a function of (sinθ)/λ (Squires, 1996 ▸; Brown, 2003 ▸). In each Bragg edge transmission corresponding to {*hkl*} diffraction, the magnetic form factor shows no wavelength dependency since Bragg’s law indicates that (sinθ)/λ is always a constant equal to 1/2*d_hkl_*.

**Figure 2:**
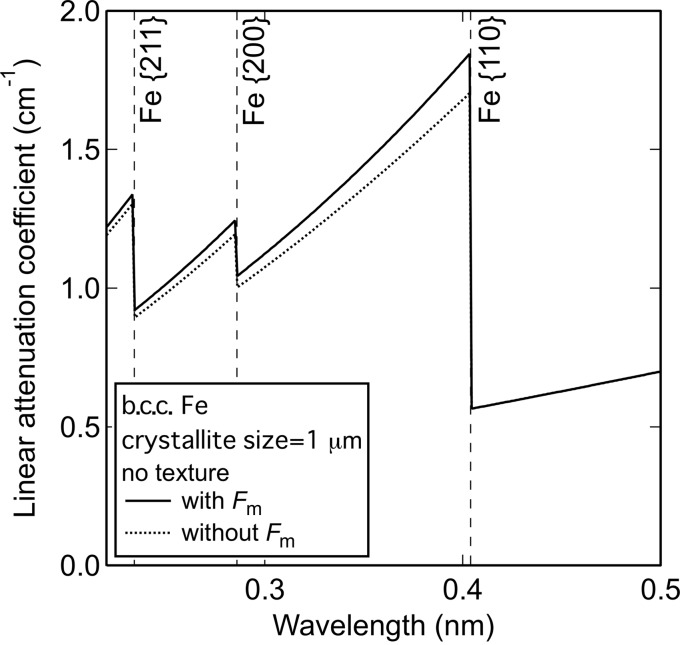
Simulated linear attenuation coefficient spectra of b.c.c. Fe. The solid and dotted curves are μ(λ) with *F*_m_ and without *F*_m_. *F*_m_ causes larger attenuation for the same crystallite size.

Fig. 2 ▸ shows μ(λ) simulated for body-centered cubic (b.c.c.) Fe with and without *F*_m_. It is assumed that the sample has no crystallographic texture, the crystallite size is 1 µm and the magnetization is 2.2 µ_B_ per atom. The jump heights of the Bragg edge transmission with *F*_m_ are significantly higher than those when only nuclear scattering was considered. This feature is similar to the effect of the change in the crystallite size (Sato *et al.*, 2011 ▸). When the crystallite becomes large, the probability of primary extinction (multiple Bragg scattering) increases; thus the intensity of the Bragg edge transmission decreases (Sabine *et al.*, 1988 ▸). If *F*_m_ is ignored in the curve fitting analysis using equation (2), a higher intensity of *F*_n_ is required to explain the height of the obtained Bragg edge transmission. Such higher intensity of *F*_n_ leads to underestimation of the crystallite size. In addition, since *F*_m_ shows θ dependence, the λ dependence of μ(λ) is affected by *F*_m_ because Bragg’s law shows that θ is related to λ. This could influence the analysis of *P_hkl_*(λ, *d_hkl_*). Thereby, if *F*_m_ is not considered, inaccurate information about the crystallographic texture is possibly obtained.

## 3. Experimental   

To experimentally investigate the effect of the magnetic scattering contribution, Fe–2 mass% Cu alloy (Cu steel) was chosen as a model steel. Since Cu steel is one of the simplest types of steel, it is frequently used in various investigations. However, the detailed precipitation process of Cu in Fe is still controversial because the morphology of Cu in Fe is highly sensitive to heat treatment conditions. One of the most important issues is concentration of Fe in the Cu precipitates. Several experimental and theoretical studies show that b.c.c. Cu can contain Fe (Goodman *et al.*, 1973 ▸; Worrall *et al.*, 1987 ▸; Zhang *et al.*, 2004 ▸). It is also argued that the precipitates can consist of several phases with different chemical compositions (Miller *et al.*, 2003 ▸). Further investigation is required to clarify the precipitation process of Cu.

To form nanometre-sized Cu precipitates, the sample was aged at 873 K for 600 s. The crystal structure of the matrix was ferrite. The magnetization of the sample was estimated to be 190 e.m.u. g^−1^ (1 e.m.u. g^−1^ = 1 A m^2^ kg^−1^) using a superconducting quantum interference device magnetometer at the CROSS-Tokai user laboratory. This is a factor of 0.9 compared with that of pure Fe and similar to what has been reported previously (Osamura *et al.*, 1993 ▸). In the present study, this value was used to calculate the magnetic scattering intensity.

The SANS and Bragg edge transmission measurements were conducted using the small- and wide-angle neutron scattering instrument BL15 TAIKAN installed at the pulsed neutron source of J-PARC (Takata *et al.*, 2015 ▸). Neutrons with a wavelength between 0.2 and 0.76 nm were used. The thickness of the sample was 1.47 mm. A magnetic field of 1 T, which is enough to saturate the magnetization of general steels, was applied using an electromagnet to separate the magnetic scattering from the nuclear scattering in SANS. Only SANS data with λ longer than 0.42 nm were converted to SANS profiles to avoid contamination by multiple Bragg diffraction. For Bragg edge transmission analysis, transmission data with λ between 0.2 and 0.55 nm were used. The Bragg edge transmissions of b.c.c. Fe {110}, {200} and {211} are included in this range. To normalize the transmission spectra with the sample volume, the transmission spectra were converted into the attenuation coefficient on the basis of the Beer–Lambert law (Oba *et al.*, 2015 ▸). To cover a wide *q* range, the SANS measurements were performed also at the SANS instrument QUOKKA installed at the Australian Nuclear Science and Technology Organisation (Gilbert *et al.*, 2006 ▸). Here, *q* is the magnitude of the scattering vector and is equal to (4π/λ)sinθ.

The SANS intensities were normalized by the sample thickness. They were also converted into absolute units using the glassy carbon standard (Zhang *et al.*, 2010 ▸) at TAIKAN and using attenuated direct beam transmission measurements at QUOKKA. To extract the scattering of only the Cu precipitates, scattering of a non-aged sample was subtracted as a background from that of the aged sample.

## 4. Results and discussion   

### 4.1. Bragg edge transmission spectra   

**Figure 3:**
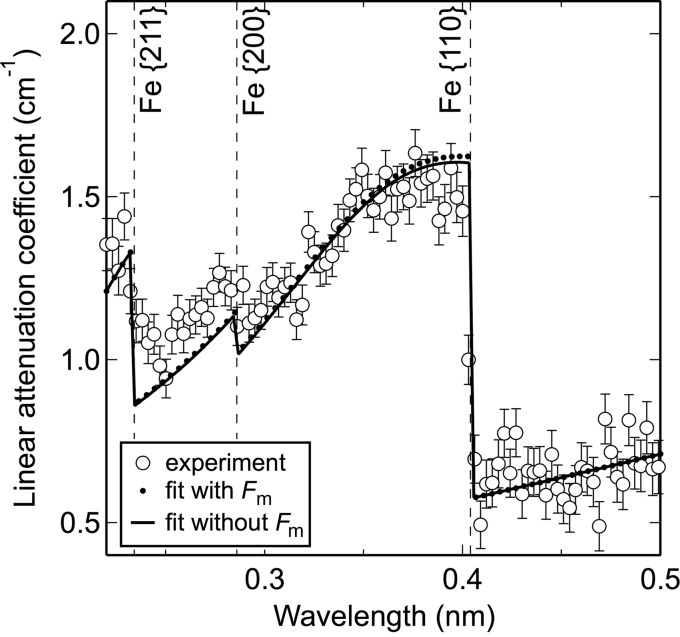
Linear attenuation coefficient spectra of the Cu steel. Vertical broken lines show the position of b.c.c. Fe Bragg edge transmissions. The dotted and solid curves are fitted curves with and without *F*_m_.

Fig. 3 ▸ shows the linear attenuation coefficient spectrum of the Cu steel. The spectrum shows clear jumps at wavelengths corresponding to the calculated Bragg edge transmission for b.c.c. Fe. The Bragg edge transmission of face-centered cubic (f.c.c.) Cu was not apparent within experimental error and no other Bragg edge transmission features were observed. Although there was a hump around λ = 0.36 nm, which corresponds to the jump by Cu {200} diffraction, the Bragg edge transmissions derived from the other lattice planes, such as {111}, were not detected. It is therefore concluded that the hump can be attributed to the texture of b.c.c. Fe and, within the experimental error, f.c.c. Cu was not observed.

Curves fitted using equation (1), both with and without *F*_m_, are shown in Fig. 3 ▸. In the present study, the lattice parameters were fixed as the values of b.c.c. Fe. The correction using the resolution function was not considered because it only affects the accurate evaluation of the lattice parameters. Both the fitted curves were in agreement with the experimental spectra; the fitted curve with *F*_m_ is seen to almost overlap that without *F*_m_.

In contrast, the crystallite sizes evaluated with and without *F*_m_ were 1.7 ± 0.1 and 1.0 ± 0.2 µm, respectively. The crystallite size obtained with *F*_m_ was significantly larger than that without *F*_m_. The difference corresponds to underestimation of the crystallite size caused by the overestimation of *F*_n_. Since the Bragg edge transmission is particularly sensitive to crystallite size in the range of 1–10 µm, these results represent an important outcome of this study (Sato *et al.*, 2011 ▸).

The *R* factors, indicating the degree of crystallographic texture (Larson & Von Dreele, 1994 ▸; Sato *et al.*, 2011 ▸), were estimated to be 0.55 ± 0.02, both with and without *F*_m_. This demonstrates that the effect of magnetic scattering on *P_hkl_*(λ, *d_hkl_*) was negligible. Although the λ dependence of the magnetic scattering can affect the fit to *P_hkl_*(λ, *d_hkl_*), the variation caused by magnetic scattering is small. Furthermore, since more than one Bragg edge transmission was overlapped when λ was shorter than 0.29 nm, this subtle λ dependence can be obscured.

### 4.2. SANS profiles   

**Figure 4:**
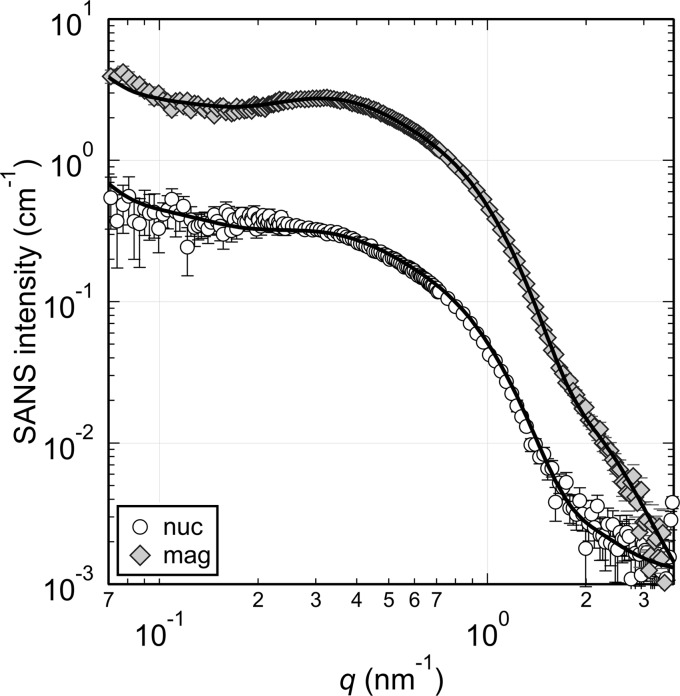
SANS profiles of the Cu steel. Open circles and grey diamonds are *I*_n_(*q*) and *I*_m_(*q*), respectively. Solid curves are the results of curve fitting.

Fig. 4 ▸ shows the SANS profiles corresponding to the Bragg edge transmission spectrum in Fig. 3 ▸. Both the magnetic *I*_m_(*q*) and nuclear scattering contribution *I*_n_(*q*) exhibit a clear shoulder, reflecting the size of the precipitates formed. The magnetic scattering profile has a gentle peak around *q* = 0.3 nm^−1^. This is attributed to the structure factor of SANS, *i.e.* the interparticle interference between the nanoparticles (Pedersen, 1994 ▸); however, the peak is not observed in *I*_n_(*q*). A difference in *q* dependence between *I*_n_(*q*) and *I*_m_(*q*) in Cu steels was also observed in previous studies (Osamura *et al.*, 1993 ▸; Miller *et al.*, 2003 ▸). This is indicative of precipitates with different scattering contrasts Δρ in the sample (Große *et al.*, 2000 ▸; Seong *et al.*, 2010 ▸); the peak observed in *I*_m_(*q*) was probably hidden in the scattering of the other precipitates in *I*_n_(*q*) because of the difference in Δρ between nuclear and magnetic scattering contributions.

To analyze the size distribution of the precipitates, curve fitting analysis was performed. The multi-phase precipitates were approximated by spherical nanoparticles with two logarithmic normal size distributions. For the structure factor of SANS, a local monodisperse approximation was used (Pedersen, 1994 ▸). In this condition, the scattering intensity *I_k_*(*q*) is given by
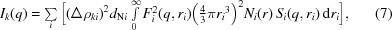
where *d*_N_ and *r* are the number density and radius of the precipitates, respectively. The subscript *k* = m or n denotes the magnetic or nuclear scattering contributions. The subscript *i* = S or L represents the phase of the small or large precipitates. The functions *F*(*q*, *r*), *N*(*r*) and *S*(*q*, *r*) are the form factor and size distribution function of precipitates, and the structure factor of the spatial distribution of precipitates, respectively. *F*(*q*, *r*) is described as
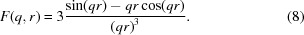
For *S*(*q*, *r*), the Percus–Yevick approximation with a hard-sphere potential was used (Pedersen, 1997 ▸). Both *I*_m_(*q*) and *I*_n_(*q*) were fitted simultaneously. The upturn of the coarse microstructures at low *q* was approximated by *q* ^−4^. The resultant curves describe both *I*_m_(*q*) and *I*_n_(*q*) well (Fig. 4 ▸) and the results of the curve fitting analysis are summarized in Table 1 ▸.

The average diameters *D*_S_ and *D*_L_ were 4.54 and 16.1 nm for phases S and L, respectively. The size *D*_S_ was similar to the diameter reported in a previous study (Geuser & Deschamps, 2012 ▸), whereas *D*_L_ is larger than that reported in the previous study. This indicates that phase L has very low visibility for neutron scattering because it was present in a much smaller amount than phase S. In individual *I*_n_(*q*) and *I*_m_(*q*) values, there was no significant feature of phase L such as a shoulder or a peak. Nevertheless, the difference between *I*_n_(*q*) and *I*_m_(*q*) is evidence for the presence of phase L.

The phases of the precipitates were subsequently determined from the results of the curve fitting. The values of Δρ_n_ reflect the mass densities and chemical compositions of the precipitates, while those of Δρ_m_ are correlated with the mass densities and magnetizations (Wiedenmann, 2000 ▸; Michels, 2014 ▸). Although these values are useful to characterize the phases of the precipitates, a usual SANS analysis gives only the product of (Δρ)^2^ and *d*_N_. However, by taking the ratio of the results fitted to *I*_m_(*q*) and *I*_n_(*q*), *d*_N_ can be eliminated and the ratio *A* = Δρ_m_^2^/Δρ_n_^2^ is obtained (Osamura *et al.*, 1993 ▸; Große *et al.*, 2000 ▸; Seong *et al.*, 2010 ▸). The values of *A* reflect the phases of the precipitates independently of the number density. The experimental values of *A*_S_ = 9.5 and *A*_L_ = 4.6 were evaluated for phases S and L, respectively (Table 1 ▸).

**Figure 5:**
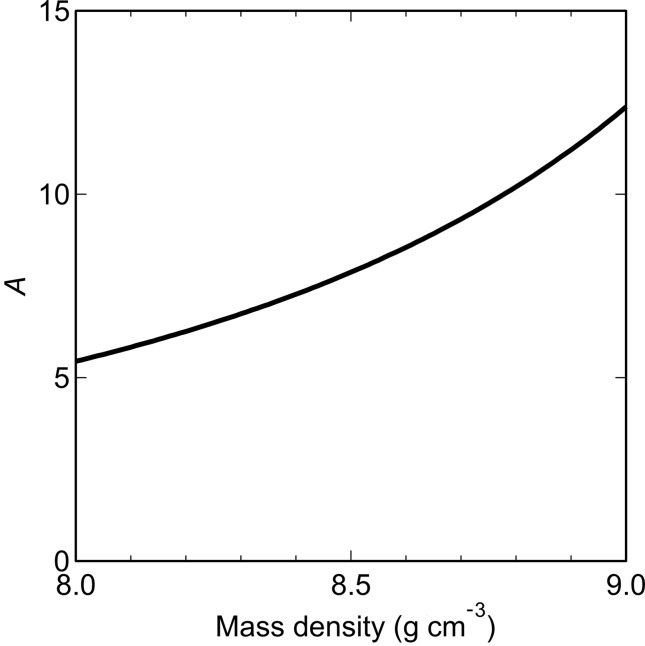
Mass density dependence of the ratio *A*. The mass densities of the b.c.c. and f.c.c. Cu are 8.1 and 8.9 g cm^−3^.

To determine the phase of the precipitates, *A* was also calculated from reference values (Ackland *et al.*, 1997 ▸). The crystal structure of Cu in Fe is known to transform from b.c.c. to f.c.c. as the size increases (Monzen *et al.*, 2000 ▸). During the transformation, hexagonal 9R and 3R structures are also formed. Although these hexagonal structures are basically close-packed ones, they can contain elastic strain and vacancies, which decrease the mass density (Monzen *et al.*, 2000 ▸; Blackstock & Ackland, 2001 ▸). Though crystallographic symmetry does not directly affect Δρ, when the crystal structures of the precipitates change, the mass densities also change. Therefore, the mass density dependence of *A* was calculated rather than the crystal structure dependence (Fig. 5 ▸). The mass densities of the b.c.c. and f.c.c. structures are 8.1 and 8.9 g cm^−3^, respectively (Ackland *et al.*, 1997 ▸). The corresponding values of *A*_b.c.c._ = 4.6 and *A*_f.c.c._ = 8.9 were calculated for the b.c.c. and f.c.c. Cu; these values are close to our experimental results *A*_L_ and *A*_S_. However, the size of phase S is smaller than that of phase L, whereas many previous studies have confirmed that the b.c.c. Cu precipitates are smaller than the f.c.c. Cu precipitates (Osamura *et al.*, 1993 ▸; Monzen *et al.*, 2000 ▸; Blackstock & Ackland, 2001 ▸). As a result, the possibility that mixing of Fe in the precipitates had occurred was considered; indeed, several researchers have pointed out that the b.c.c. Cu precipitates can contain Fe (Goodman *et al.*, 1973 ▸; Worrall *et al.*, 1987 ▸; Zhang *et al.*, 2004 ▸).

**Figure 6:**
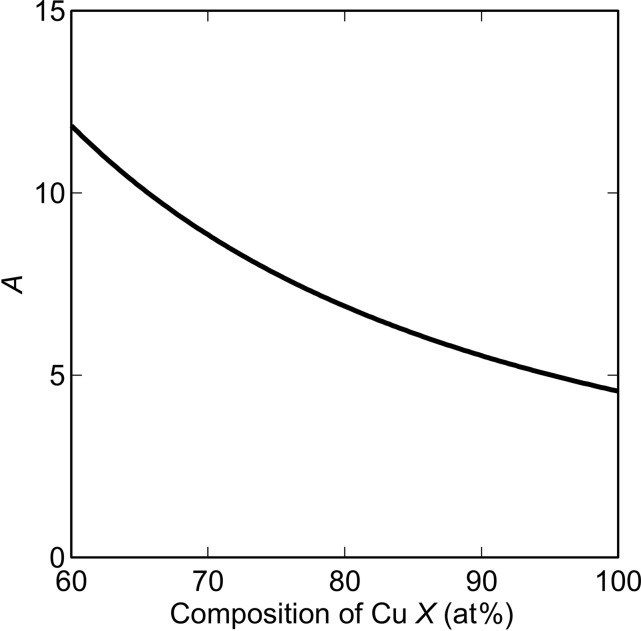
Chemical composition dependence of *A. X* is the Cu content in the b.c.c. Cu*_X_*Fe_1−*X*_.

Fig. 6 ▸ shows the calculated values of *A* as a function of the Cu content *X* in the b.c.c. Cu*_X_*Fe_1−*X*_ alloy based on previous SANS studies (Osamura *et al.*, 1993 ▸; Miller *et al.*, 2003 ▸). By comparing the experimental and calculated values, phase S was determined to be Cu_0.7_Fe_0.3_. Previous studies have reported that *X* changes approximately between 0.5 and 1 in Cu steel (Goodman *et al.*, 1973 ▸; Worrall *et al.*, 1987 ▸). In addition, Miller *et al.* (2003 ▸) showed two possibilities for *X*: *X* = 0.98, 2% vacancies and no Fe, or *X* = 0.72, 8% vacancies and 20% Fe. The current value of *X* = 0.7 is therefore comparable to that of previous studies; the variation in *X* between these studies is probably caused by differences in the aging conditions.

On the other hand, the experimental value of *A*_L_ matched the calculated value of *A*_b.c.c._. However, according to the previous studies, the b.c.c. to 9R transformation occurs when the precipitate size is between 4 and 12 nm (Monzen *et al.*, 2000 ▸). The average diameter *D*_L_ was larger than this reported critical size. Phase L is therefore likely to be the 9R structure with elastic strain and vacancies, the latter resulting in a decrease in the mass density. It is noted that the SANS results cannot distinguish between b.c.c. and strained 9R structures if the mass density is the same.

Consequently, the transformation mechanism of Cu precipitates can be summarized as follows. With the aging of Cu steel, b.c.c. Cu–Fe is first precipitated. With increasing size, Fe in the precipitates moves to the matrix leaving elastic strain and vacancies. During this precipitation process, the crystal structure probably transforms from b.c.c. to 9R.

## 5. Conclusion   

The magnetic scattering contribution to simultaneous SANS and Bragg edge transmission measurements has been investigated and applied to Cu steel. The expression for the magnetic scattering contribution to Bragg edge transmission has also been outlined. In the Bragg edge transmission analysis, the crystallite size determined by including the magnetic scattering contribution was larger than that conventionally reported in its absence. This indicates that the magnetic scattering contribution has to be considered to perform an accurate evaluation of the crystallite size. In the SANS analysis, comparison of the magnetic and nuclear scattering contributions allowed the chemical composition of the precipitates to be determined. The nanoparticles were composed of b.c.c. Fe–Cu and pure Cu. The crystal structure of the pure Cu precipitates is proposed to have the 9R structure with elastic strain and vacancies.

## Figures and Tables

**Table 1:** Average diameter *D* and ratio *A* of magnetic to nuclear scattering evaluated from curve fitting

Phase	*D* (nm)	*A*
Phase S	4.54 ± 0.02	9.51 ± 0.04
Phase L	16.1 ± 0.6	4.6 ± 0.3

## References

[bb1] Ackland, G. J., Bacon, D. J., Calder, A. F. & Harry, T. (1997). *Philos. Mag. A*, **75**, 713–732.

[bb2] Blackstock, J. J. & Ackland, G. J. (2001). *Philos. Mag. A*, **81**, 2127–2148.

[bb3] Brown, P. J. (2003). *Neutron Data Booklet*, 2nd ed., edited by A.-J. Dianoux & G. Lander, p. 2.5-1. Philadelphia: Old City Publishing.

[bb4] De Geuser, F. & Deschamps, A. (2012). *C. R. Phys.* **13**, 246–256.

[bb5] Gilbert, E. P., Schulz, J. C. & Noakes, T. J. (2006). *Phys. B Condens. Matter*, **385–386**, 1180–1182.

[bb6] Goodman, S. R., Brenner, S. S. & Low, J. R. (1973). *Metall. Trans.* **4**, 2371–2378.

[bb7] Granada, J. R. (1984). *Z. Naturforsch. Teil A*, **39**, 1160–1167.

[bb8] Große, M., Gokhman, A. & Böhmert, J. (2000). *Nucl. Instrum. Methods Phys. Res. Sect. B*, **160**, 515–520.

[bb9] Harjo, S., Tomota, Y., Lukáš, P., Neov, D., Vrána, M., Mikula, P. & Ono, M. (2001). *Acta Mater.* **49**, 2471–2479.

[bb10] Heenan, R. K., Penfold, J. & King, S. M. (1997). *J. Appl. Cryst.* **30**, 1140–1147.

[bb11] Larson, A. C. & Von Dreele, R. B. (1994). *GSAS*. Report LAUR 86-748. Los Alamos National Laboratory, New Mexico, USA.

[bb12] Michels, A. (2014). *J. Phys. Condens. Matter*, **26**, 383201. 10.1088/0953-8984/26/38/383201 25180625

[bb13] Miller, M. K., Wirth, B. D. & Odette, G. R. (2003). *Mater. Sci. Eng. A*, **353**, 133–139.

[bb14] Monzen, R., Jenkins, M. L. & Sutton, A. P. (2000). *Philos. Mag. A*, **80**, 711–723.

[bb15] Oba, Y., Morooka, S., Sato, H., Sato, N., Ohishi, K., Suzuki, J. & Sugiyama, M. (2015). *ISIJ Int.* **55**, 2618–2623.

[bb16] Ohnuma, M., Suzuki, J., Ohtsuka, S., Kim, S.-W., Kaito, T., Inoue, M. & Kitazawa, H. (2009). *Acta Mater.* **57**, 5571–5581.

[bb17] Osamura, K., Okuda, H., Takashima, M., Asano, K. & Furusaka, M. (1993). *Mater. Trans. JIM*, **34**, 305–311.

[bb18] Pedersen, J. S. (1994). *J. Appl. Cryst.* **27**, 595–608.

[bb19] Pedersen, J. S. (1997). *Adv. Colloid Interface Sci.* **70**, 171–210.

[bb20] Sabine, T. M., Von Dreele, R. B. & Jørgensen, J.-E. (1988). *Acta Cryst.* A**44**, 374–379.

[bb21] Sato, H., Kamiyama, T. & Kiyanagi, Y. (2011). *Mater. Trans.* **52**, 1294–1302.

[bb23] Seong, B. S., Shin, E., Choi, S.-H., Choi, Y., Han, Y. S., Lee, K. H. & Tomota, Y. (2010). *Appl. Phys. A*, **99**, 613–620.

[bb24] Squires, G. L. (1996). *Introduction to the Theory of Thermal Neutron Scattering.* New York: Dover Publications.

[bb25] Takata, S., Suzuki, J., Shinohara, T., Oku, T., Tominaga, T., Ohishi, K., Iwase, H., Nakatani, T., Inamura, Y., Ito, T., Suzuya, K., Aizawa, K., Arai, M., Otomo, T. & Sugiyama, M. (2015). *JPS Conf. Proc.* **8**, 036020.

[bb26] Tomota, Y., Lukáš, P., Neov, D., Harjo, S. & Abe, Y. R. (2003). *Acta Mater.* **51**, 805–817.

[bb27] Wiedenmann, A. (2000). *J. Appl. Cryst.* **33**, 428–432.

[bb28] Worrall, G. M., Buswell, J. T., English, C. A., Hetherington, M. G. & Smith, G. D. W. (1987). *J. Nucl. Mater.* **148**, 107–114.

[bb29] Zhang, C., Enomoto, M., Yamashita, T. & Sano, N. (2004). *Met. Mater. Trans. A*, **35**, 1263–1272.

[bb30] Zhang, F., Ilavsky, J., Long, G. G., Quintana, P. G., Allen, A. J. & Jemian, P. R. (2010). *Met. Mater. Trans. A*, **41**, 1151–1158.

